# Primary Cardiac Angiosarcoma with Rare Presenting Feature and Successful Surgical Treatment

**DOI:** 10.21470/1678-9741-2017-0239

**Published:** 2018

**Authors:** Arzu Antal Donmez, Davut Cekmecelioglu, Taylan Adademir, Ekrem Yilmaz, Hizir Mete Alp

**Affiliations:** 1 Department of Cardiovascular Surgery, Kartal Kosuyolu Heart Research and Training Hospital, Istanbul, Turkey.

**Keywords:** Heart Neoplasms, Hemangiosarcoma, Neoplasm Metastasis, Cardiovascular Surgical Procedures, Heart Atria

## Abstract

Primary angiosarcoma is a rare clinical entity, it's typically located within the right atrium and known to be rapidly fatal. A 37-year-old female was presented with a history of recurrent facial paralysis and left hemiparesis. A cranial mass was identified at cranial magnetic resonance imaging and she underwent neurosurgery operation. The immunohistochemical examination was determined as metastatic cardiac angiosarcoma. The tumor, as well as part of the right pericardium, were resected. A piece of bovine pericardium was used to reconstruct the right atrial wall. Tricuspid valve was reconstructed with ring annuloplasty. Due to resection of right coronary artery with the tumor, coronary bypass surgery was performed successfully. The patient is currently healthful without any recurrence and complaint 12 months after the diagnosis as followed up.

**Table:** 

Abbreviations, acronyms & symbols	
CPB	= Cardiopulmonary bypass
CT	= Computed tomography
ICU	= Intensive care unit
MR	= Magnetic resonance

## INTRODUCTION

While primary malignant cardiac tumors, including the pericardium, are rare, angiosarcomas are the most commonly reported histologic type. Primary angiosarcoma is also a rare clinical entity, with an incidence of 0.0001% in collected autopsy series^[1,2]^. It's typically located within the right atrium as large symptomatic masses and known to be rapidly fatal, with the diagnoses usually determined only at autopsy. It frequently extends to the pericardium, vena cava, or tricuspid valve, causing tamponade and/or heart inflow obstruction^[2]^. Early heart transplantation and novel radiation therapy approaches may offer a survival benefit in non-metastatic tumors, but up to 80% of the patients present with systemic metastasis at diagnosis^[1]^. Metastases are common, and often widespread. Clinical diagnosis of angiosarcoma is often difficult because there are no specific symptoms associated with the disease. Symptoms can be either cardiac or systemic. The majority of the literature describes a uniformly dismal prognosis with a median survival of only 6 months for these aggressive tumors^[3,4]^. Additionally, with the aid of newer imaging techniques, localization, biopsy diagnosis and resection of the atrial tumors are now being achieved more often, with some improvement in survival.

Complete surgical resection confers the best long-term outcome but may be contraindicated because of anatomic considerations in advanced tumors^[5]^. We report a patient with a right atrial angiosarcoma which first presented clinically as a cerebral tumor mimicking a glioma.

## CASE REPORT

**Fig. 1:**
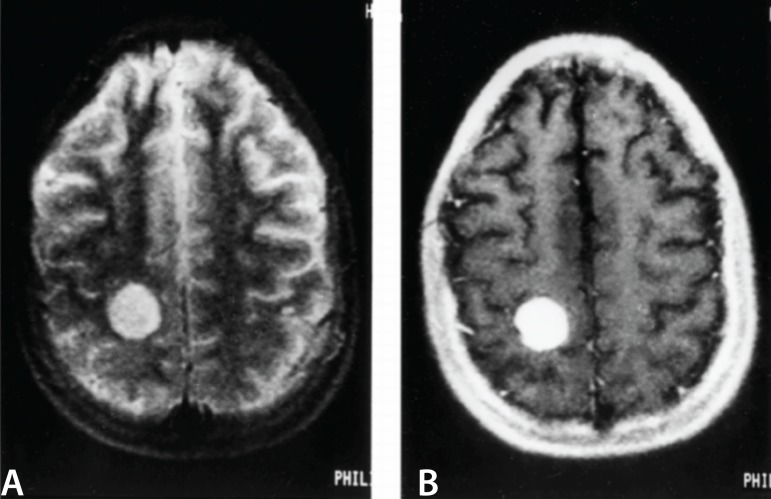
The lesion in the right parietal lobe hyperintense on the long MRI image (A) and hypointense on short TR image, showed marked contrast enhancement (B). The lesion grew on several studies and was a surgically proven to be hemangiosarcoma.

**Fig. 2:**
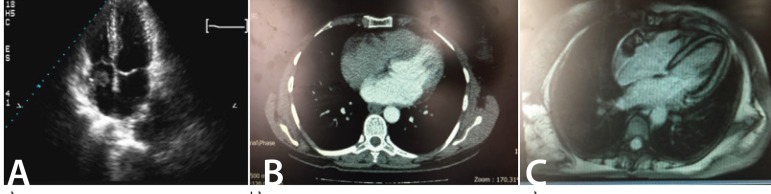
The pathology of the resected tumor from parietal lobe confirms angiosarcoma with vasoformative architectures. H&E × 200

A 47-year-old female was presented to a private hospital with a history of recurrent facial paralysis and left hemiparesis. The patient had no other pertinent past medical history. After a marked right-sided visual field defect developed, various investigations disclosed a tumor in the right parieto-occipital region. A cranial mass was identified at cranial magnetic resonance (MR) imaging (Figure 1) and she underwent neurosurgery operation. It was surgically resected and found to be a 3-cm spherical red mass with central necrosis, sharply demarcated from the surrounding brain tissue by a thin condensation of fibrous tissue. It was composed entirely of well-formed, blood-filled, anastomosing sinusoidal-type spaces formed by branching trabeculae of loose fibrocellular stroma covered by an endothelial cell lining, generally single-layered with occasional doubling (Figure 2). Mitoses were quite uncommon. This lesion was reported as a hemangioma, although the atypical location and histologic features were noted. These immunohistochemical examination was determined as metastatic angiosarcoma. She also got adjuvant chemotherapy. The regimen of the therapy administered by medical oncology department was doxorubicin (75 mg/m^2^) and ifosfamide (7500 mg/m^2^) with a duration of 6 cycles of combination treatment.

**Fig. 3:**
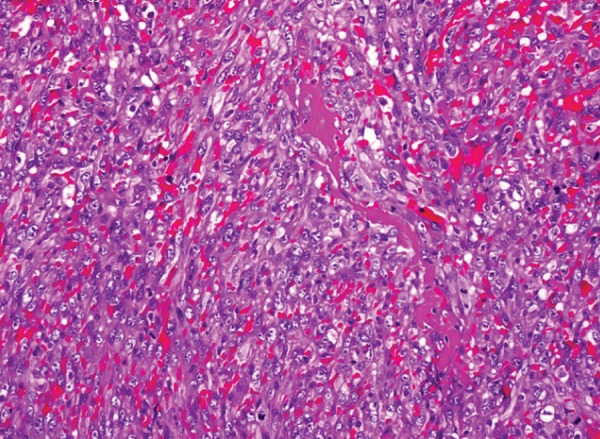
Preoperative cardiac images with different screening methods. A) transesophageal echocardiography, B) computed tomography, C) cardiac magnetic resonance imaging.

About 6 weeks after neurosurgery, she reappeared with shortness of breath and pericardial friction rub but no increased jugular venous pressure or dependent edema. She was transferred to our institution and underwent transesophageal echocardiography, which showed a homogeneous mass that involved the free wall of the right atrium. The patient then underwent computed tomography (CT), MR imaging, and angiography (Figure 3). Findings from the mass suggested a diagnosis of cardiac angiosarcoma as 3x3 cm. Electrocardiogram showed normal sinus rhythm with nonspecific ST wave changes. Chest X-ray revealed cardiomegaly and a pericardial effusion, confirmed by echocardiography. An abdominal ultrasound showed only an enlarged liver. By evaluation of our heart team, we decided to proceed with complete surgical resection of primary tumor.

**Fig. 4:**
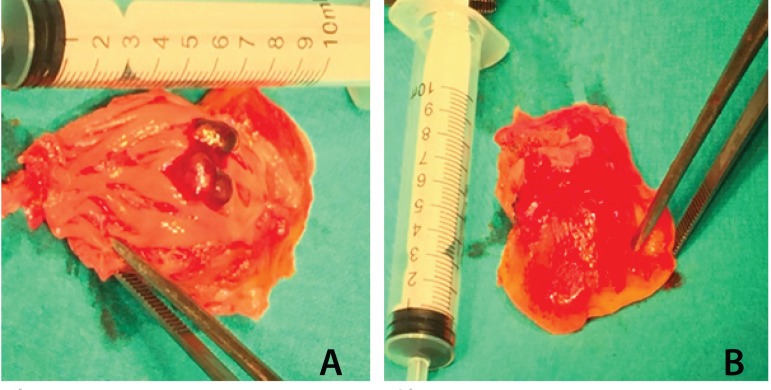
Resected primary cardiac angiosarcoma intraoperative images. A) inferior wall of right atrium reveals with 3x3 cm tumoral mass, B) outer surface of the right atrial wall with stuck pericardium.

**Fig. 5:**
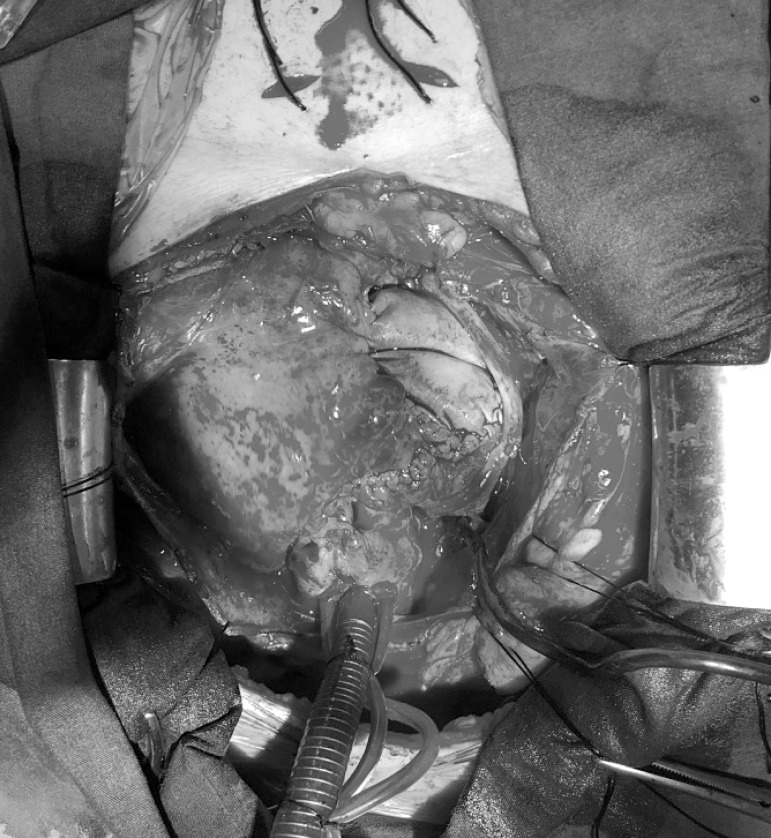
Image of patch plasty of right atrium and vein graft to the right coronary artery at the end of surgery.

Full-arterial monitoring was performed under general anesthesia. Standard aortic arterial and bicaval venous cannulation were performed. Cardiopulmonary bypass (CPB) was achieved. Cardiac arrest was achieved with blood cardioplegia. After cross-clamping, surgical field was exposed with right atriotomy. The tumor, as well as part of the right pericardium, were resected (Figure 4). Because of tumor involvement of anterosuperior leaflet, tricuspid valve was reconstructed with ring annuloplasty. A piece of bovine pericardium was used to reconstruct the right atrial wall via continue suture technique. And due to resection of the right coronary artery with the tumor, distal anastomosis of coronary bypass surgery with saphenous vein was performed (Figure 5). Proximal anastomosis was performed with side clamped as well. She has weaned from the CPB smoothly. Heparin neutralization with protamine was performed.

Aortic cross-clamp time was 36 minutes and total perfusion time was 65 minutes. During the operation, mean arterial pressure was maintained at 70 mmHg. By the end of CBP, the patient was transferred to intensive care unit (ICU) with a hemodynamically stable condition. She was extubated on the postoperative 7^th^ hour.

Total drainage was 500 cc. After one night staying in ICU, the patient was discharged in good condition after the 7^th^ day of the operation. Follow up echocardiographic controls revealed clean cardiac cavities. The control routine biochemistry was normal and 9^th^ month PET scan was also clean.

In the present report, a patient with cardiac angiosarcoma and distant metastases was treated with a combination of chemotherapy and surgical resections. The patient responded well to neoadjuvant chemotherapy, and the tumor and metastases decreased to an extent that made complete surgical resection feasible. Surgical resection was successful in removing the mass and subsequent chemotherapy was used to destroy any remaining tumor cells.

## DISCUSSION

Even up to 80% of the patients present with systemic metastasis at diagnosis, and in the case reported on this article primary cardiac angiosarcoma with rare manifestation with cerebral metastasis was treated successfully with neoadjuvant and adjuvant chemotherapy and complete surgical resection of the tumor with the reconstruction of the right atrium. With the correct combination of multidisciplinary treatments for cardiac angiosarcoma, we hope that a cure for this disease is possible rather than treatment regimens being used as either palliative or life-prolonging measures.

While other authors have published case reports of long-term survival after multidisciplinary treatment for angiosarcoma, these were patients presented without metastases^[6]^. This patient was presented with several systemic metastases, but with the combination of neoadjuvant and adjuvant chemotherapy and complete surgical resection, the patient is currently alive and without recurrence 12 months after diagnosis with functional class New York Heart Association grade I. Multidisciplinary approach is mandatory to improve long-term survival.

Although this patient has not experienced recurrence, this is an unusual case and diligence in the surveillance for new tumor growth is, therefore, imperative.

**Table:** 

Authors' roles & responsibilities	
AAD	Substantial contributions to the conception or design of the work; or the acquisition, analysis, or interpretation of data for the work; final approval of the version to be published
DC	Drafting the work or revising it critically for important intellectual content; final approval of the version to be published
TA	Final approval of the version to be published; final approval of the version to be published
EY	Agreement to be accountable for all aspects of the work in ensuring that questions related to the accuracy or integrity of any part of the work are appropriately investigated and resolved; final approval of the version to be published
HMA	Agreement to be accountable for all aspects of the work in ensuring that questions related to the accuracy or integrity of any part of the work are appropriately investigated and resolved; final approval of the version to be published
